# Unsustainability of Obesity: Metabolic Food Waste

**DOI:** 10.3389/fnut.2016.00040

**Published:** 2016-10-07

**Authors:** Mauro Serafini, Elisabetta Toti

**Affiliations:** ^1^Functional Foods and Metabolic Stress Prevention Laboratory, Centre for Food and Nutrition, Council for Agricultural Research and Economics, Rome, Italy

**Keywords:** sustainable nutrition, obesity, metabolic food waste, functional diet, ecological footprints, inflammation, human, animal products

## Abstract

The obesity burden, with 1.5 billion overweight (OW) and 500 million obese (OB) worldwide, significantly increased the risk of degenerative diseases. Excessive consumption of foods that are energy dense lead to obesity, which represents a titanic cost for not only the world’s health systems but also a substantial ecological cost to the environment. The waste of resources and the unnecessary green house gas emissions (GHGs) emission, due to “obesigen” consumption of foods, have been ignored so far in practical assessments of ecological impacts. Our position is that food eaten above physiological needs, manifesting as obesity, should be considered waste. In this study, we developed a new indicator, metabolic food waste [MFW_(kg of food)_], corresponding to the amount of food leading to excess body fat and its impact on environment expressed as carbon $\left[{\text{MFW}}_{\text{(kg}{\text{CO}}_{2}\text{eq)}}\right]$, water [MFW_(×10 L)_], and land footprint $\left[{\text{MFW}}_{\left(\times 10{\text{m}}^{2}\right)}\right]$. Results shows that the average amount of MFW_(kg of food)_ was of 63.1 and 127.2 kg/capita in a observational study on 60 OW and OB subjects. Animal products contributed mostly to MFW_(kg of food)_ in both OW (24.3 kg) and OB (46.5 kg), followed by cereals, legumes and starchy roots (19.4 kg OW; 38.9 kg OB), sugar and sweets (9.0 kg OW; 16.4 kg OB), and alcoholic beverages (7.5 kg OW; 20.1 kg OB). When dietary intake corresponding to MFW was transformed in ecological indexes, animal products displayed the highest values for carbon emissions, water consumption, and land use in both OW and OB followed by cereals, legumes, and starchy roots. The estimated MFW_(kg of food)_ of the Italian population resulted to be 2.081 million kilograms of food for OB and OW. Reducing obesity will make a contribution toward achieving sustainable and functional diets, preserving and re-allocating natural resources for fighting hunger and malnutrition, and reducing GHGs emissions. Although further evidences in epidemiological studies are needed, MFW represents an innovative and reliable tool to unravel the diet–environment–health trilemma.

## Introduction

During the last decade, there has been significant public, scientific, and political awareness raised about the importance of following a sustainable dietary pattern, optimizing agriculture food chains, and limiting food loss and waste (FLW) to protect the environment (1). Food losses represent the decrease in edible food mass throughout the part of the supply chain that specifically leads to edible food for human consumption and take place at production; postharvest and processing stages while food waste involve retailers and consumers behavior (2, 3). Total FLW globally have been estimated at 1.3 billion tons per year, roughly one-third of the food produced for human consumption, leading to a remarkable waste of natural resources and a massive amount of green house gas emissions (GHGs), which negatively affect climate change (4). Recently, the data on ecological footprints, such as water, carbon, and land use, have allowed the assessment of the impact of single foods and dietary pattern on the environment in terms of resources and GHGs (5). The majority of the evidence clearly shows that, for the same amount of food, animal products such as fish and meat require considerable natural resources and are among the highest contributors to GHGs emission, differently from food of vegetable origins characterized by a lower ecological impact (6).

The obesity burden, with 1.5 billion overweight (OW) and 500 million obese (OB) worldwide (7), has serious implications for health, significantly increasing the risk of cardiovascular diseases, type 2 diabetes, and certain type of cancers. The chronic consumption of nutritionally unbalanced meals, often characterized by emphasis on excessive amount of sugars and fats from meats, dairy products, fried foods, processed snack foods, and sweets, may result in various postprandial metabolic stressors that are detrimental for the cardiovascular system, as well as to an increased release into the circulation of reactive oxygen species, pro-inflammatory cytokines, and adhesion molecules leading to an exacerbated immune response of the body (8–11). On the contrary, a high intake of food of vegetable origin, due to the high content of functional ingredients and to the low energy load, can efficiently counteract postprandial stress (8, 12). The obesity condition, other than being an excessive fat deposit, is characterized by an excessive and uncontrolled cytokines production, a condition defined as “low-grade chronic inflammation” associated with the development of degenerative diseases (13, 14). In this context, the importance of the diet, as inducer or preventer of obesity, is paramount for maintaining physiological homeostasis and preserving health.

Excessive consumption of energy dense foods leading to obesity represents a titanic cost for not only the world’s health systems but also a substantial ecological cost to the environment. Dietary patterns higher in refined sugars, fats, oils, and meat have been shown to be the major contributor to about 80% increase in GHGs from food production, challenging the diet–environment–health triangle (6). Individual dietary patterns regarding food choice, nutrient, and phytochemical content are tightly linked to nutritional sustainability and environmental protection (15–17), underpinning the concept that Planet Health cannot be detached from Human Health. The waste of resources and the unnecessary GHGs emission due to an excessive consumption of foods leading to obesity and inflammatory conditions have been ignored so far in quantitative assessments of ecological impacts.

Our position is that food eaten above physiological needs, manifesting as obesity, should be considered as waste. Here, we developed a new indicator, metabolic food waste [MFW_(kg of food)_], corresponding to the amount of food leading to excess body fat (EBF) and its impact on environment expressed as carbon $\left[{\text{MFW}}_{\text{(kg}{\text{CO}}_{2}\text{eq)}}\right]$, water [MFW_(×10 L)_], and land footprint $\left[{\text{MFW}}_{\left(\times 10{\text{m}}^{2}\right)}\right]$. MFW_s_ from OW and OB people were measured in an observational study and estimated in the Italian population.

## Materials and Methods

Approximately 30 OW and 30 OB subjects were randomly selected from an internal database including participants of other surveys and intervention trials carried out from our research group. Subjects were recruited on the basis of their BMI (>25 kg/m^2^), absence of illness or any pathologies, not taking supplements, no intense or moderate physical activity, consumption of less than four portion of fruits and vegetables for a week, omnivores, not vegan, macrobiotic, or vegetarian. All subjects gave written informed consent in accordance with the Declaration of Helsinki.

Dietary intake was assessed by means of a validated 4-day recall record questionnaire, three consecutive working days, and one weekend day or holiday as fully described by Willett (18). The subjects were asked to record the amounts of food consumed by food weighting or with the help of visual tools in order to increase the accuracy of portion size. Dietitians checked all completed records and dietary patterns that were calculated in terms of foods. Foods were aggregated on the basis of similar macronutrient composition into five commodities: cereals, starchy roots, and legumes; added fats; animal products (fish and meat); sugar/sweets; and alcoholic beverages. Fruits and vegetables were excluded because they are low energy dense foods not contributing to obesity. The difference among individual and average of BMI range for normal-weight people (21.75 kg/m^2^) was multiplied for energy content of 1 kg of body fat (32.2 MJ) to reach the total energy from EBF and distributed among the different foods according to their percentage contribution to total energy intake. The acquired data allowed to calculate MFW_(kg of food)_, carbon ${\text{MFW}}_{\text{(kg}{\text{CO}}_{2}\text{eq)}}$, water MFW_(×10 L)_, and land ${\text{MFW}}_{\left(\times 10{\text{m}}^{2}\right)}$ (19, 20).

In order to estimate MFW of the Italian population, the data of the individual diets have been applied to the Italian OW and OB population (21). The number of OW and OB individuals was extracted from the WHO Global Database on body mass index (22). EBF, difference between average BMI from normal-weight and OW or OB subjects, was multiplied for the energy content of 1 kg of body fat. The MFW_(kg of food)_, carbon ${\text{MFW}}_{\left(\text{kg}{\text{CO}}_{2}\text{eq}\right)}$, water ${\text{MFW}}_{\left({\text{m}}^{2}\right)}$, and land ${\text{MFW}}_{\left({\text{m}}^{2}\right)}$ were calculated as described above for individual subjects.

## Results

**Figure 1:**
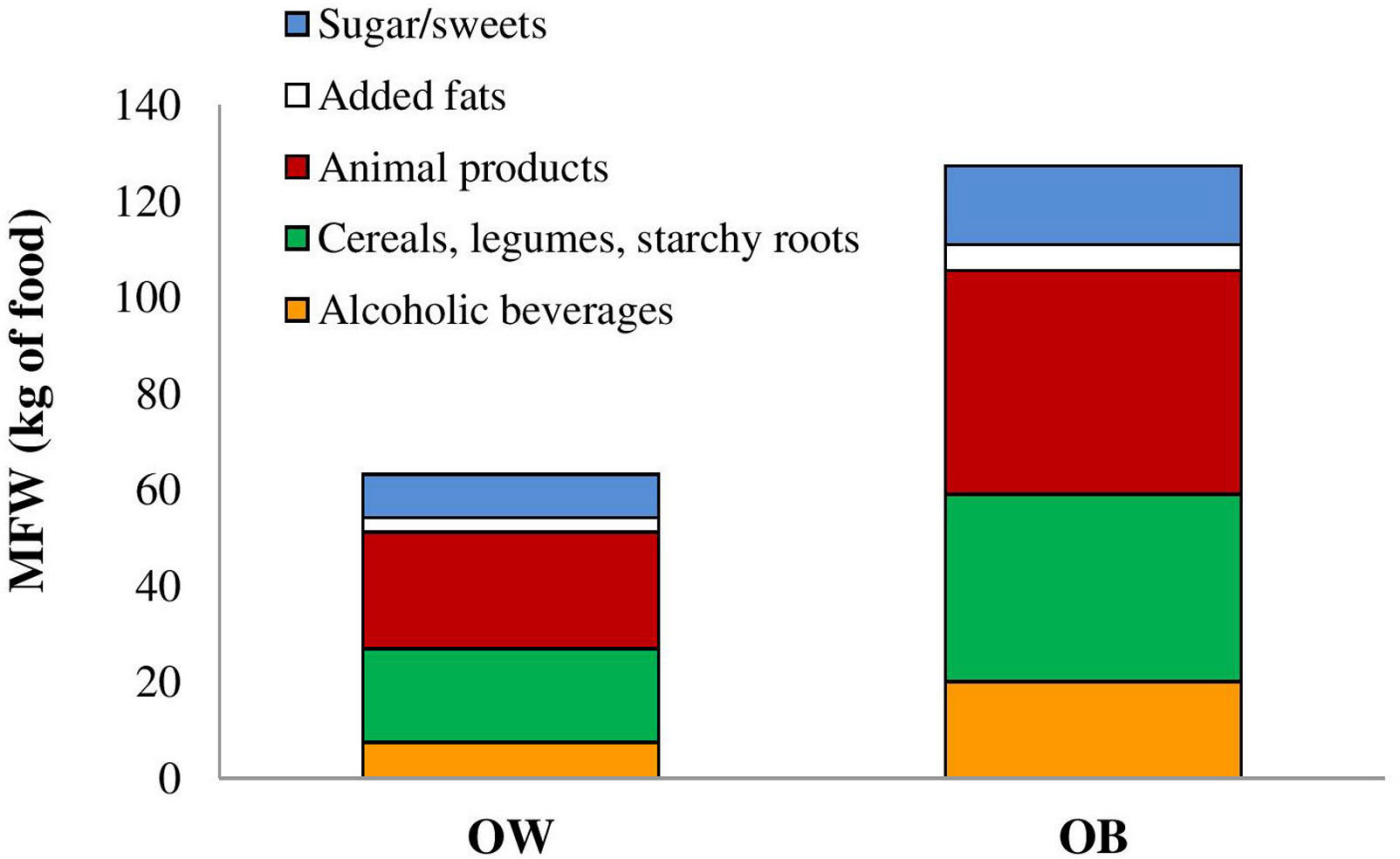
**Metabolic food waste corresponding to excess body fat from food commodities in overweight (OW) and obese (OB) subjects expressed as amount of food [MFW_(kg of food)_]**.

**Figure 2:**
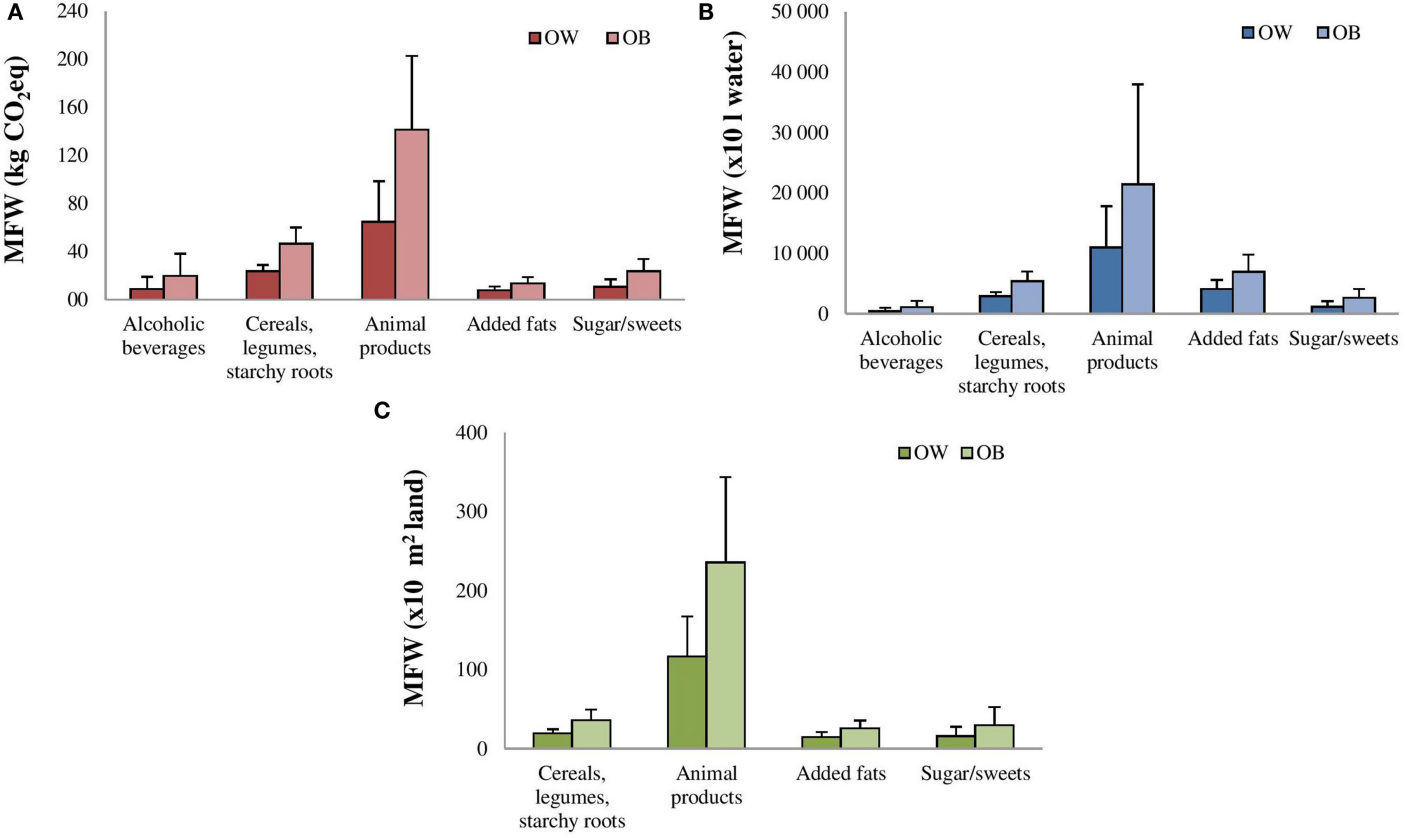
**Metabolic food waste corresponding to excess body fat from food commodities in overweight (OW) and obese (OB) subjects expressed as (A) GHGs emission, ${\text{MFW}}_{\left(\text{kg}{\text{CO}}_{2}\text{eq}\right)}$; (B) water consumed, MFW_(×10 L water)_; (C) land used, ${\text{MFW}}_{\left(\times 10{\text{m}}^{2}\text{ land}\right)}$**.

The physical characteristics and the EBF expressed as kilograms and kilojoules for OW and OB subjects are displayed in Table 1. Average MFW_(kg of food)_ corresponding to EBF was of 63.1 and 127.2 kg/capita, respectively, for OW and OB subjects, as described in Figure 1. Animal products contributed mostly to MFW_(kg of food)_ in both OW (24.3 kg) and OB (46.5 kg), followed by cereals, legumes and starchy roots (19.4 kg OW; 38.9 kg OB), sugar and sweets (9.0 kg OW; 16.4 kg OB), and alcoholic beverages (7.5 kg OW; 20.1 kg OB). When dietary intake corresponding to MFW was transformed in ecological indexes, animal products displayed the highest values for carbon emissions, water consumption, and land use in both OW and OB (Figures 2A–C) followed by cereals, legumes, and starchy roots. As overall the amount of food corresponding to EBF and its impact on the environment from the 60 subjects was 5.710 MFW_(kg of food)_, 10.794 ${\text{MFW}}_{\left(\text{kg}{\text{CO}}_{2}\text{eq}\right)}$, 1711.492 MFW_(×10 L water)_, and 14.821 ${\text{MFW}}_{\left(\times 10{\text{m}}^{2}\text{ land}\right)}$.

**Table 1:** **Physical characteristics of the subjects: results are expressed as mean ± SD for overweight (OW) and obese (OB)**.

		OW	OB
**Age**	years	37.8 ± 11.9	40.5 ± 12.4
**Gender**	M/F	14/16	13/17
**Weight**	kg	77.7 ± 8.8	99.4 ± 9.9
**Height**	cm	167.3 ± 8.2	173.6 ± 7.2
**BMI**	kg/m^2^	27.7 ± 0.7	32.9 ± 1.4
**EBF**	kg	16.9 ± 3.2	33.9 ± 5.6
	kJ	542,695 ± 103,632	1,090,587 ± 181,482

**Figure 3:**
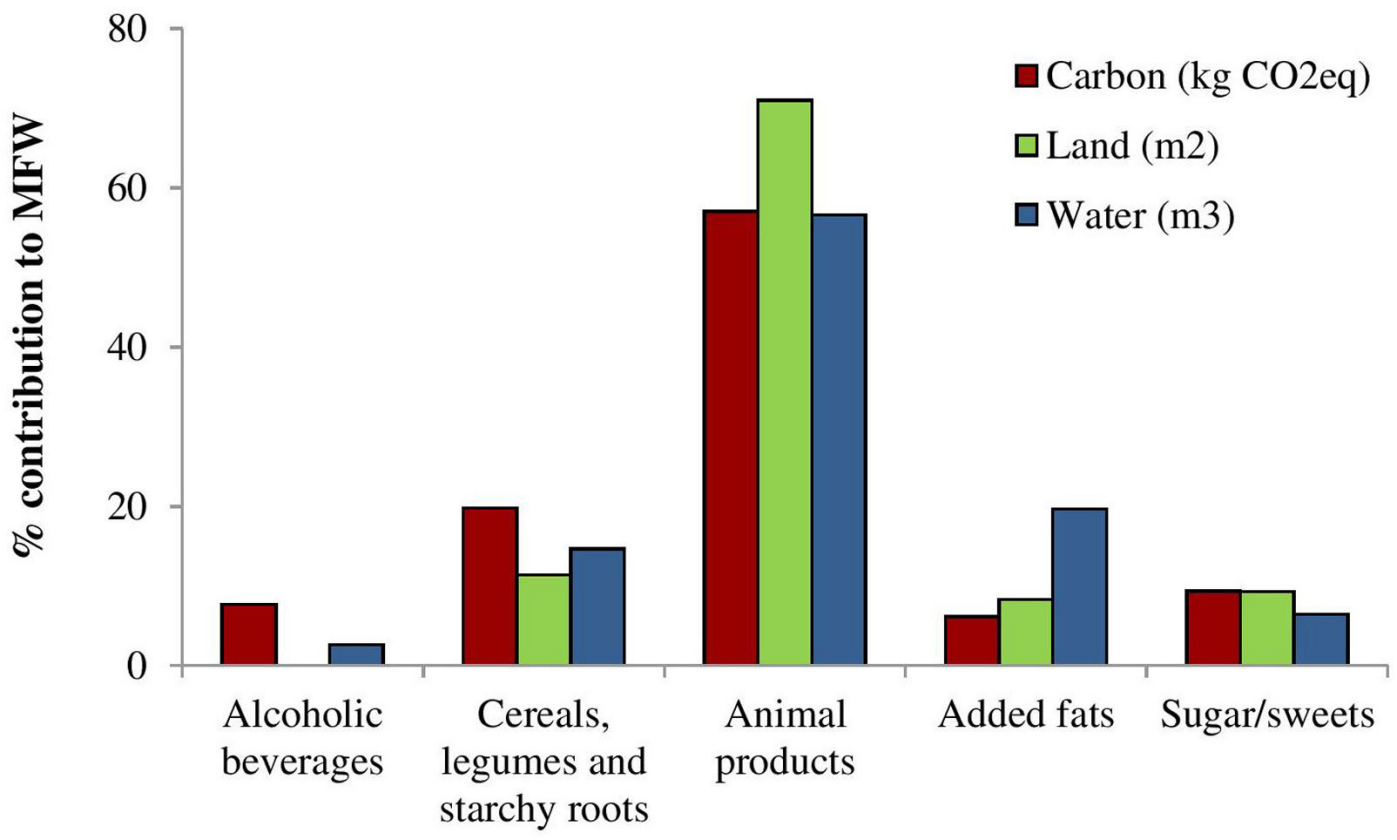
**Percentage contribution of GHGs emission, 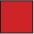
${\text{MFW}}_{\left(\text{kg}{\text{CO}}_{2}\text{eq}\right)}$; water consumed, 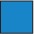
${\text{MFW}}_{\left({\text{m}}^{3}\text{ water}\right)}$; land used, 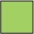
${\text{MFW}}_{\left({\text{m}}^{2}\text{ land}\right)}$ to total MFW from food commodities in overweight and obese Italian population**.

The estimated MFW_(kg of food)_ of the Italian population resulted to be 1.319 million kilograms for OW and 762 million kilograms of food wasted as EBF for a total of 2.081 million kilograms of food as reported in Table 2. Ecological footprints corresponding to EBF were of 2.409 and 1.466 million ${\text{MFW}}_{\left(\text{kg}{\text{CO}}_{2}\text{eq}\right)}$ for GHGs emissions; 4.090 and 2.246 million ${\text{MFW}}_{\left({\text{m}}^{3}\text{ water}\right)}$ for water footprint and of 34,858 and 19,612 million ${\text{MFW}}_{\left({\text{m}}^{2}\text{ land}\right)}$ for land footprint, respectively, for OW and OB. Animal products were the highest contributor to the three MFW ecological footprints, with a 57, 71, and 57% of the total, respectively, for carbon, land, and water footprints followed by cereals, legumes and starchy roots, sugar sweets for GHGs emission, and land used, as displayed in Figure 3.

**Table 2:** **Estimated metabolic food waste in overweight (OW) and obese (OB) Italian**.

MFW	OW	OB
Food (million kg)	1319	762
Water (million m^3^)	4090	2246
Carbon (million kg CO_2_eq)	2409	1466
Land (million m^2^)	34,858	19,612

## Discussion

Understanding the link between diet and its impact on human and planet health represent a global challenge for the scientific community as well as for Government and individuals. The double role of diet as inducer or preventer of obesity is tightly linked to its impact on the environment and utilization of resources. In this paper, we suggest that the food eaten above physiological needs, manifesting as obesity and leading to an increased risk of degenerative diseases, represents a significant waste of food as well as of the natural resources utilized; producing unnecessary GHGs emission deleterious for the environment. We developed for the first time a quantitative indicator, MFW_(kg of food)_, corresponding to the amount of food leading to EBF and its impact on environment expressed as carbon $\left[{\text{MFW}}_{\text{(kg}{\text{CO}}_{2}\text{eq)}}\right]$, water [MFW_(×10 L)_], and land footprint $\left[{\text{MFW}}_{\left(\times 10{\text{m}}^{2}\right)}\right]$. In a observational study on 60 OW and OB subjects, we have shown that the average amount of MFW_(kg of food)_ was of 63.1 and 127.2 kg/capita, with the main contributor coming from meat and fish. When we estimate the MFW_(kg of food)_ in Italian population, the total value was 2.081 million kilograms of food wasted in obesity status. These figures suggest the massive amount of food and ecological resources as well as production of GHGs emissions potentially wasted by the 2 billion people in the world who are OB and OW.

As expected and in agreement with previous evidences (5), meat and fish represent the main contributor to MFW and to the waste of resources and increase in GHGs emissions in our subjects. However, there are some points that need to be raised; first of all, we suffer for a lack of information about the ecological footprints of “obesigen” foods such as soft drinks, snacks, and junk food, limiting the amount of food to include in the assessment of MFW. Under this point of view, it is highly recommendable that ecological footprints database will be amplified and improved for number of items in order to identify exactly the individual contribution of single foods without any approximation. Meat and fish in our figure represent the major determinants of MFW and of its ecological impact. Red meat and sausages have a very high ecological footprint and have been associated with a higher risk of degenerative diseases (23, 24); however, this is not valid for fish, which contains functional ingredients such as omega-3 and phytochemicals that can be beneficial for obesity prevention. The assessment of ecological impact cannot be the only determinant to define recommendation for adequate intake, but it should also substantiate and be merged by information on nutritional and phytochemical values, assessing the ecological cost of production and functional benefit for the body.

Enriching the diet with more food of vegetable origin represents a winning strategy under the health and the ecological point of view. Fruits and vegetables have been widely shown to protect against degenerative disease for their high content in vitamins, antioxidant, and bioactive ingredients. Moreover, the association of plant food during a high stressor meal deeply reduces the inflammatory/oxidative load and the increase of metabolic risk factors (25). At the same time, plant foods are characterized by lower ecological footprints respect to animal foods, guaranteeing a double effect on metabolic and environmental protection. However, we need to keep in mind that the five portions a day of fruits and vegetables recommended for a healthy dietary pattern might provide more ecological expenditure than a limited weekly amount of meat. This is a focal point that is still missing in the literature and highlights the importance to assess ecological impact through proper dietary weekly intake data and not just with a simple comparison with the same amount. This approach will allow adjusting the intake of specific foods on the basis of ecological, nutritional, and functional point of view.

Fat accumulation and obesity development is a day-to-day process, related to lifestyle, dietary choices, physical activity, gut microbiota, hormones, etc., during the entire life of the subjects and might change during different ages and conditions, following that the MFW we calculated is the result of a much higher amount of unnecessary food leading to the actual impaired metabolic condition. In this view, assessing MFW in epidemiological studies will allow to monitor the raise of obesity associated with unbalanced dietary regimen and calculating the impact on environment during time. One flaw in our estimate of MFW in the Italian population, an issue that we share with the rest of scientific community working on food waste, is the lack of reliable database and proper information. Data from FBS cannot be considered intake data, and for this reasons, we did not utilize it; moreover, it does not take into consideration all single food items in each commodity and, as can be easily highlighted, there are voices like “other meat” including the wide variety available in nature but without any specific information. This bias is also shared with the figures so far published on FLW extracted from the Global Food Losses and Food Waste report (2) where detailed single food items are missing, making necessary developing detailed database in order to properly calculate MFW and FLW.

In conclusion, MFW represents an innovative and reliable tool to unravel the diet–environment–health trilemma; providing for the first time a figure for the massive amount of food lost through obesity, amplifying statistics on global food waste, and showing that obesity is an “unsustainable” metabolic condition.

## Author Contributions

MS had the original idea to calculate MFW and wrote the manuscript. ET extracted and analyzed the data, and finalized the calculations of MFW. Both authors discussed the results and implications and commented on the manuscript at all stages.

## Conflict of Interest Statement

The authors declare that the research was conducted in the absence of any commercial or financial relationships that could be construed as a potential conflict of interest.
